# Luminescence of colloidal CdSe/ZnS nanoparticles: high sensitivity to solvent phase transitions

**DOI:** 10.1186/1556-276X-6-142

**Published:** 2011-02-14

**Authors:** Andrei Antipov, Matt Bell, Mesut Yasar, Vladimir Mitin, William Scharmach, Mark Swihart, Aleksandr Verevkin, Andrei Sergeev

**Affiliations:** 1 Electrical Engineering Department, University at Buffalo, Buffalo, NY 14260, USA; 2 Chemical and Biological Engineering Department, University at Buffalo, Buffalo, NY 14260, USA

## Abstract

We investigate nanosecond photoluminescence processes in colloidal core/shell CdSe/ZnS nanoparticles dissolved in water and found strong sensitivity of luminescence to the solvent state. Several pronounced changes have been observed in the narrow temperature interval near the water melting point. First of all, the luminescence intensity substantially (approximately 50%) increases near the transition. In a large temperature scale, the energy peak of the photoluminescence decreases with temperature due to temperature dependence of the energy gap. Near the melting point, the peak shows N-type dependence with the maximal changes of approximately 30 meV. The line width increases with temperature and also shows N-type dependence near the melting point. The observed effects are associated with the reconstruction of ligands near the ice/water phase transition.

## 

Optical methods for the characterization of phase transitions have attracted attention of many research groups as sensitive, rapid, and extremely effective technique which responds to small changes in crystallographic structures, stress and local lattice distortions, changes in stoichiometry, and dislocations [1-3]. Variety of luminescence techniques such as thermoluminescence, electroluminescence, cathodoluminescence, X-ray irradiation, and ion beam luminescence can be used for excitation of luminescence [4]. A phase transition in bulk inevitably alters luminescence spectra, line widths, efficiency of excitation and recombination, excited state lifetimes, and polarization of emission bands. On the other hand, the unique photoluminescence (PL) properties of colloidal semiconductor nanoparticles (NPs) [5-7] with minimal surface functionalization have potential not only as imaging agents but also as local nanosensors due to their high sensitivity to local environment. For example, CdSe NPs placed in polymer matrix demonstrate significant changes in their temperature-dependent PL intensity and maximum PL spectral shifts. This phenomenon can potentially be used for optical probing of local temperature at nanoscale distances [8]. There are also numerous reports [9,10], which show significant influences of the surface chemistry on optical properties of colloidal NPs due to their large surface-to-volume ratios. However, the real processes can be much more complicated because NPs are partially covered by capping molecules depending on its shape, size, and surface quality of NPs [10].

In this study, we demonstrate high sensitivity of PL of colloidal NPs to the solvent state. In a series of measurements, we investigate the PL properties of CdSe/ZnS core/shell colloidal nanoparticles dissolved in water in the temperature range of 230-300 K. We also study the dry CdSe Core nanoparticles for comparison.

The control *dry *colloidal NPs sample is prepared by a spin coating of a dilute solution of 5.6-nm-diameter CdSe NPs on clean glass cover slips. *In-liquid *samples are prepared by loading a highly diluted solution of the same core-shell CdSe/ZnS NPs in water into a vacuum-sealed low-temperature optical cell. In this optical cell, the solution is held between two epitaxially polished sapphire windows separated by a 0.5-mm-thick indium foil spacer. Each sample is then mounted inside a helium continuous-flow cryostat for low-temperature optical measurements over the temperature range of *T *= 10-300 K with temperature controlled to better than 0.5 K. The input window in cryostat was diffuse quartz, which is completely transparent for the visible spectrum. To avoid any possible oxidation of samples, they are isolated in the pumped cryostat immediately after preparation and measured. The NPs are excited by a *λ *= 532 nm Nd-vanadate laser with pulse repetition rate of 76 MHz and 7 ps pulse duration. The photoluminescence from NPs is collected by a home-built confocal microscope and delivered to a 0.75-m-long imaging monochromator coupled with a single-photon sensitive electron-multiplication CCD camera. The photoluminescence from a sample is filtered by long-pass 550-nm filter, which absorbs scattering light from a pump beam.

The PL intensity of dry CdSe colloidal NPs as a function of temperature and wavelength is shown in Figure 1a. The integrated emission intensity (integration is done within λ = 550-650 nm range) slightly decreases as the temperature increases from 10 K up to 70 K. Then, at higher temperatures, it quenches dramatically in the temperature range of *T *= 70-300 K and exhibits exponential behavior. We did not observe any significant changes in PL over that temperature range, except very slow oscillation in PL tail. It is important to notice that the saturation of PL intensity observed in our experiment at the temperatures below 50 K is certainly related to the pulse repetition rate of the laser (12.5 ns) because the low-temperature radiative lifetime of the exciton can achieve an unusually long recombination time of 1 μs at very low temperatures below 10 K and the stronger dependence of PL intensity can be expected in experiments with low repetition rate excitation [7].

**Figure 1:**
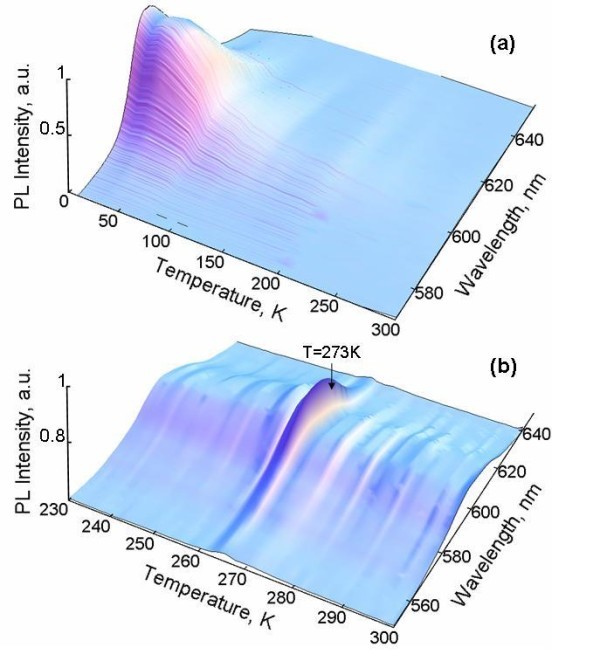
**PL intensity. of dry (a) and in-liquid colloidal (b) CdSe/ZnS NPs as functions of temperature and wavelength.)(color online)**.

Photoluminescence of the in-liquid sample dramatically differs from dry NPs behavior and exhibits several local peaks at some distinct temperatures in the temperature range of 230-300 K. The most pronounced local maximum in PL intensity (approximately 50%) occurs near the water freezing point *T *= 273 K (Figure 1b). However, the temperature position of this maximum is shifted by about 5 K below the expected phase transition temperature (see Figure 2).

**Figure 2:**
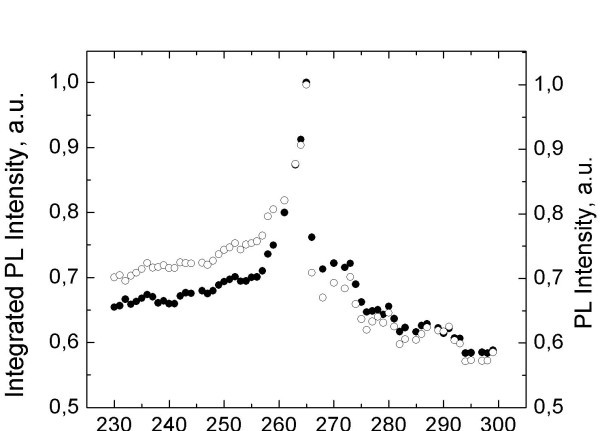
**Integrated PL intensity (solid circles) and PL peak intensity (open circles) of in-liquid CdSe/ZnS NPs**.

PL peak energy of in-liquid and dry colloidal CdSe/ZnS NPs in the temperature range of *T *= 240-290 K are shown on Figure 3. In-liquid CdSe/ZnS NPs are near the water freezing point. The dashed and solid lines are the best-fit curves to Varshni relation for dry and in-liquid NPs, respectively. It is clearly seen that PL peak energy of in-liquid NPs exhibits not only the monotonic temperature dependence similar to dry NPs sample but the N-type feature near the solvent phase transition. The PL peak energy increases by approximately 30 meV, from approximately 2.07 eV to approximately 2.1 eV, as the temperature changes from 260 to 270 K. Also, PL peak energy at low and high temperatures decreases at practically the same rate with increasing temperature.

**Figure 3:**
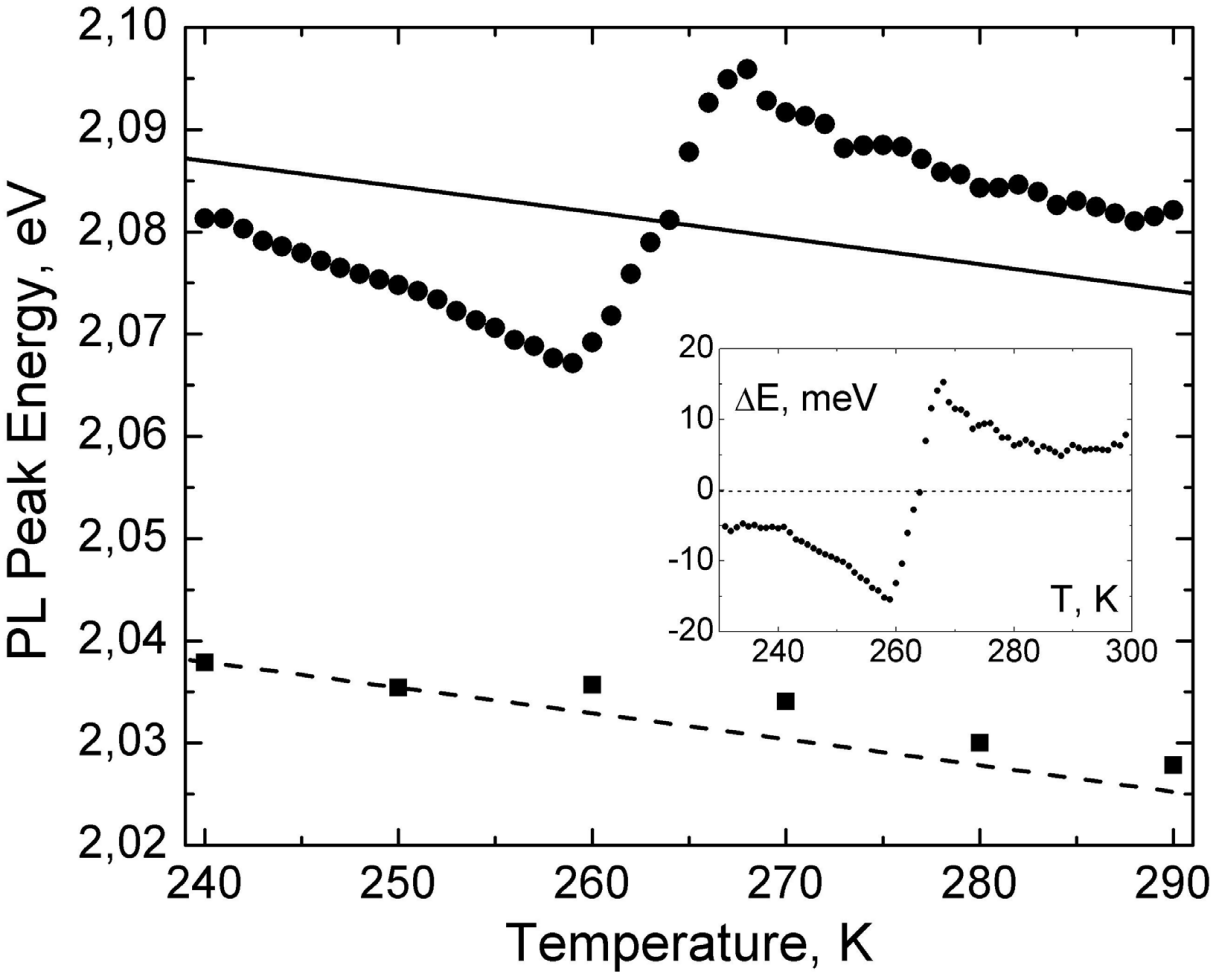
**PL peak energy of (squares) dry colloidal CdSe NPs sample and (circles) in-liquid CdSe/ZnS NPs**. The insert shows the same dependence for in-liquid NPs without monotonic part introduced in Equation 1.

PL full width at half maximum (FWHM) for in-liquid CdSe/ZnS NPs in the temperature range of *T *= 240-290 K is shown on Figure 4. Another feature is observed near the water freezing point. The FWHM increases by approximately 40 meV, from approximately 0.12 eV to approximately 0.16 eV, as the temperature changes from 260 to 270 K. However, PL shows substantially different behavior at low and high temperatures. The FWHM decreases much faster in the temperature range *T *= 270-290 K than that at *T *= 240-260 K. Also, it is important to notice that the FWHM for dry NPs does not show peculiarities within the temperature range *T *= 240-290 K.

**Figure 4:**
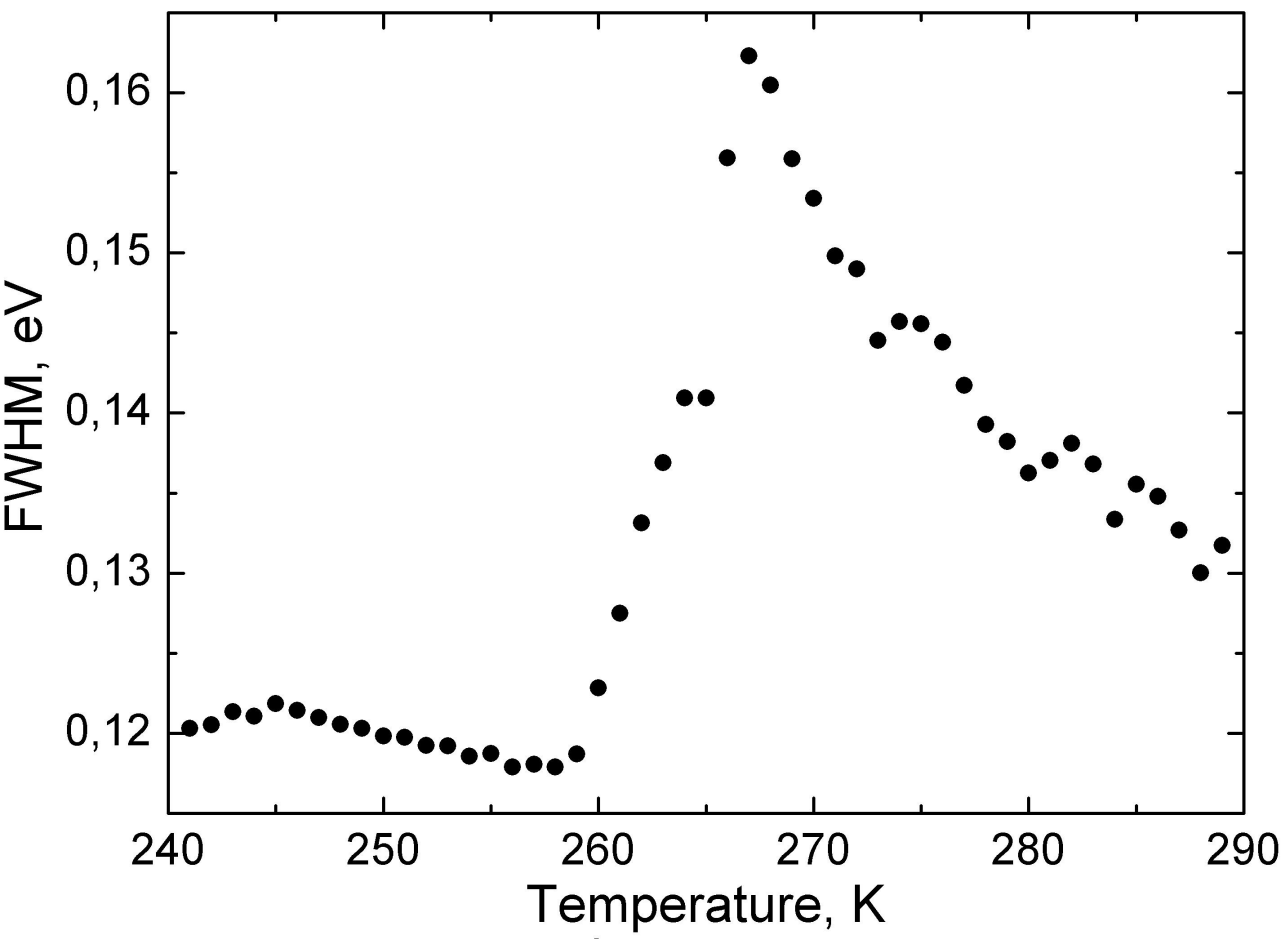
**PL FWHM of in-liquid CdSe/ZnS NPs near the water freezing point**.

We also investigate the temperature dependence of exciton lifetime of in-liquid CdSe/ZnS NPs near the water freezing point. Time-resolved measurements are performed using the time-correlated single-photon counting system, PicoHarp 300. PL decay curves are analyzed by multiexponential fitting. As it is shown in the insert of Figure 5, PL response consists of two (fast and slow) exponential components. The fast component of PL decay at *T *= 240-290 K is shown in Figure 5. It undergoes the shift by approximately 200 ps, from 150 to 350 ps, within a temperature range of 260-270 K. The fast component decreases in the temperature range *T *= 240-260 K and slowly increases at *T *= 260-290 K. The slow component of PL decay curve does not exhibit any changes in the temperature range *T *= 240-290 K and stays the same for approximately 10 ns. The experimental investigations of dry NPs show that there are no changes in exciton lifetime as for the slow component and for the fast component of PL decay curve within the temperature range *T *= 240-290 K. New N-type feature that we report here correlates very well with the behavior of exciton lifetime of in-liquid NPs near the water freezing point.

**Figure 5:**
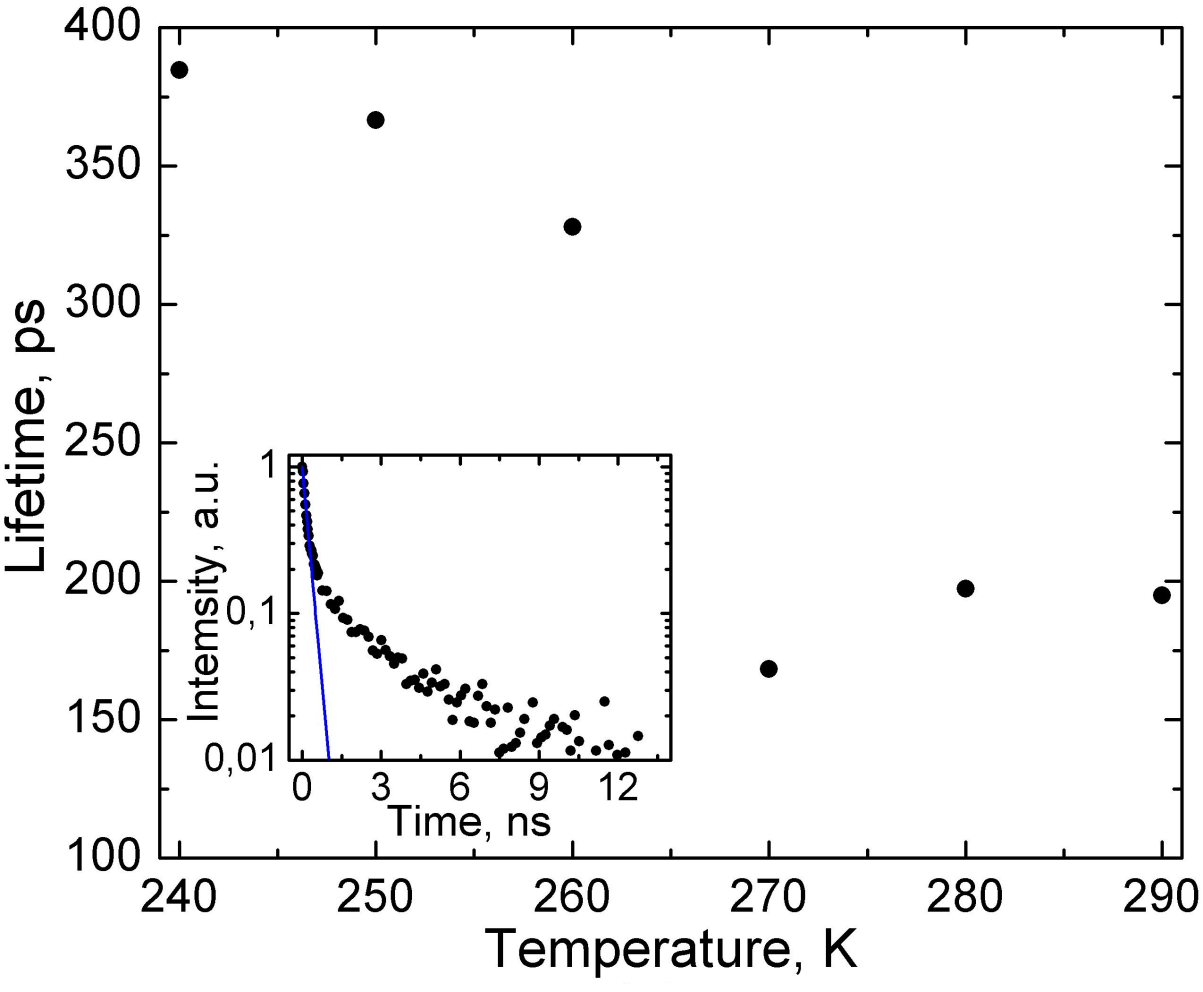
**Exciton lifetime of in-liquid CdSe/ZnS NPs near the water freezing point**. The insert shows the fit (solid line) to the fast component of PL decay curve.

We now discuss the above observed features in PL behavior of in-liquid colloidal NPs. First, we exclude possible external pressure effects during freezing. Kim et al. [11] observed increase of photoluminescence peak energy with pressure for dilute dispersions of CdSe nanocrystals in toluene or 4-ethyl pyridine and attributed this to the pressure dependences of the bulk CdSe band gap and confinement energies. Similarly, in water dispersed CdSe/ZnS NPs, we can expect some changes in pressure near the water freezing point. In our experiment, the sample was sealed between two sapphire windows that limit expansion upon freezing. However, our data show an opposite sign of the effect, the PL peak energy red shifts while the water is getting frozen in contrast to the blue shift shown in Figure 3. Most likely, the actual changes of the bulk CdSe band gap and the electron and hole confinement energies are negligibly small within the temperature range from 260 to 270 K.

Next, we can exclude the possibility of solvent freezing-point depression by addition of the NPs [12]. The estimated freezing-point depression of the dispersion prepared by adding CdSe/ZnS NPs at the concentration used here is about 10^-4 ^K. It should be noticed that all measurements are carried out at elevating temperature. One of the reasons for this is that the freezing temperature shows hysteresis, which is observed in our experiment, and can be overcooled by decreasing temperature. Another reason is difficulties related to controlling of liquid helium flow in the cryostat with the temperature controller. Also, all features in PL measurements are reproducible.

Also, papers [13] and [14] have shown a decrease of PL peak energy for water-soluble CdTe QD around 270 K as the temperature increases over a very narrow range (less than 10 K). They attribute this phenomenon to a strong influence of solid-liquid phase transition in the capping molecules on the size-dependent "luminescence temperature antiquenching" [13,14]. This, however, is opposite to our experimental result. The behavior of PL peak energy exhibits the blue shift as temperature increases from 260 to 270 K.

Our results for PL intensity and peak energy of dry colloidal NPs confirm the recent reports by different groups [15,16]. In a large temperature scale *T *= 20-300 K, the energy peak of the photoluminescence decreases with temperature due to temperature dependence of the energy gap [17]. The empirical Varshni relation [18] describes the temperature dependence of the effective band gap of bulk semiconductors:

(1) $$E_{\text{g}}(T)=E_{\text{g}}(0)-\frac{\alpha T^{2}}{\beta +T},$$

where *E*_g_(0) is the energy gap at 0 K, *α *is the temperature coefficient, and *β *is the Debye's temperature parameter of the semiconductor. The best-fit curve (Figure 3) gives *E*_g_(0) = 2.08 and 2.13 eV for dry (dashed line) and in-liquid (solid line) NPs, respectively. The different values for the energy gap can be explained by the slight difference in size of NPs. The temperature coefficient *α *= 3.2 × 10^-4 ^eV/K and the Debye's temperature *β *= 220 K are close to the values known in the literature for bulk CdSe [11].

The insert in Figure 3 represents the result of subtraction of the Varshni relation (Equation 1) from the experimental data of PL peak energy for in-liquid NPs. It shows the non-monotonic N-type dependence and can be attributed to additional mechanisms on the surface of NPs near the melting point.

We associate the observed effects with the reconstruction of surface/ligands near the ice/water phase transition. The numerous experimental results [19,20] show that effects related to surface relaxation/reconstruction, dangling bonds, and capping ligands depend on particular functionalization of NPs. Currently, it is well understood that capping molecules (ligands), which are intentionally formed on surface of NPs during their synthesis, change substantially surface properties of NPs. The formation of ligands is necessary because they prevent the aggregation of colloidal nanoparticles. Also, they control their dispersibility in solvents as well as allowing bioconjugation. Another advantage of ligands is surface passivation, i.e., reduction of the amount of Cd or Se surface dangling bonds, which creates nonradiative channels of electron-hole pair recombination. For instance, passivation of surface defects and intrinsic energy states suppresses these channels and leads to increasing of NP's quantum yield. Hence, the water phase transition can influence the surface properties of NPs directly through ligands. Deformations in the capping layer change the positions of surface states and move them out from the band gap [13]. These changes, in turn, may influence mechanisms of radiative recombination of electron-hole pairs through surface states.

In conclusion, we have demonstrated characteristic peculiarities in the PL behavior of in-liquid colloidal CdSe/ZnS nanoparticles near the water phase transition (*T *= 273 K). Several pronounced features in photoluminescence peak energy and line width of up to approximately 25 meV are observed. Both the peak energy and line width undergo the blue shift to higher energies while the solvent is melting. Those features are not observed in dry samples made with the same NPs.

## Competing interests

The authors declare that they have no competing interests.

## Authors' contributions

AA, MB, and MY made PL measurements; WS and MS carried out synthesis and characterization of nanoparticles; VM, AV, and AS planned and analyzed experiments, developed the model, and together with AA prepared the manuscript. All authors approved the final version of the manuscript.
